# Pharmacogenetic association between *NAT2* gene polymorphisms and isoniazid induced hepatotoxicity: trial sequence meta-analysis as evidence

**DOI:** 10.1042/BSR20180845

**Published:** 2019-01-15

**Authors:** Saif Khan, Raju K. Mandal, Abdulbaset Mohamed Elasbali, Sajad A. Dar, Arshad Jawed, Mohd Wahid, Harishankar Mahto, Mohtashim Lohani, Bhartendu Nath Mishra, Naseem Akhter, Ali A. Rabaan, Shafiul Haque

**Affiliations:** 1 Department of Basic Science, College of Dental Sciences, University of Ha’il, Ha’il 2440, Saudi Arabia; 2 Research and Scientific Studies Unit, College of Nursing and Allied Health Sciences, Jazan University, Jazan 45142, Saudi Arabia; 3 Department of Clinical Laboratory Science, College of Applied Medical Sciences, Al Jouf University, Sakakah 72388, Saudi Arabia; 4 Centre for Life Sciences, Central University of Jharkhand, Ranchi 835205, Jharkhand, India; 5 Department of Emergency Medical Services, College Applied Medical Sciences, Jazan University, Jazan 45142, Saudi Arabia; 6 Department of Biotechnology, Institute of Engineering and Technology, Lucknow 226021, Uttar Pradesh, India; 7 Department of Laboratory Medicine, Faculty of Applied Medical Sciences, Albaha University, Albaha 65431, Saudi Arabia; 8 Molecular Diagnostic Laboratory, John Hopkins Aramco Healthcare, Dhahran 31311, Saudi Arabia

**Keywords:** anti-tuberculosis drug, genetic model, hepatotoxicity, Meta-analysis, NAT2

## Abstract

Hepatotoxicity is a severe problem generally faced by tuberculosis (TB) patients. It is a well-known adverse reaction due to anti-TB drugs in TB patients undergoing long-term treatment. The studies published previously have explored the connection of N-acetyltransferase 2 (*NAT2*) gene polymorphisms with isoniazid-induced hepatotoxicity, but the results obtained were inconsistent and inconclusive. A comprehensive trial sequence meta-analysis was conducted employing 12 studies comprising 3613 controls and 933 confirmed TB cases using the databases namely, EMBASE, PubMed (Medline) and Google Scholar till December 2017. A significant association was observed with individuals carrying variant allele at position 481C>T (T vs. C: *P* = 0.001; OR = 1.278, 95% CI = 1.1100–1.484), at position 590G>A (A vs. G: *P* = 0.002; OR = 1.421, 95% CI = 1.137–1.776) and at position 857G>A (A vs. G: *P* = 0.0022; OR = 1.411, 95% CI = 1.052–1.894) to higher risk of hepatotoxicity vis-à-vis wild-type allele. Likewise, the other genetic models of *NAT2* gene polymorphisms have also shown increased risk of hepatotoxicity. No evidence of publication bias was observed. These results suggest that genetic variants of *NAT2* gene have significant role in isoniazid induced hepatotoxicity. Thus, *NAT2* genotyping has the potential to improve the understanding of the drug–enzyme metabolic capacity and help in early predisposition of isoniazid-induced hepatotoxicity.

## Introduction

*Mycobacterium tuberculosis* is the single most prominent species responsible for tuberculosis (TB) disease. This is a commonly prevalent disease present in almost all societies of the world. It causes a significant burden of morbidity and mortality on human population. The data from the year 2015 showed that almost 10.4 million people suffered from TB leading to 1.8 million deaths (http://www.who.int/mediacentre/factsheets/fs104/en/). The current most effective control is curing the patients with anti-TB drugs like isoniazid or isonicotinylhydrazide (INH), rifampicin (RMP) and pyrazinamide (PZA). These anti-TB drugs are used in combination for 6 or more months to cure the infection completely. The most common side effect associated with the use of anti-TB drugs is idiosyncratic hepatotoxicity, worldwide [1]. Hepatotoxicity occurs with greater frequency in patients receiving both INH and RMP vis-à-vis patients treated with isoniazid alone [2]. Predicting the occurrence of anti-TB drug therapy induced hepatotoxicity is difficult, but it has been observed that its variation in the patients is between 1 and 36% [3]. It has been observed that many patients are at higher risk of developing hepatotoxicity during the course of anti-TB chemotherapy than others [3]. This individual susceptibility could be associated with the genetic variation in the gene(s) involved in the drug metabolism, transport or excretion, or gene(s) that regulates the immune responses [4]. Thus, elucidating the genetics involved in anti-TB drugs like INH-induced hepatotoxicity would be of clinical significance and may provide valuable information to clinicians as well as patients interested in continuing the therapy.

The most vital organ in human body for drug metabolism is liver. It also helps in detoxification of the body. Being very active, it is more vulnerable to injury as well. Liver metabolizes INH to acetylisoniazid with the help of N-acetyltransferase 2 (*NAT2*). The acetylisoniazid generated is then hydrolyzed to acetylhydrazine that is disposed after acetylation by *NAT2* to form a non-toxic metabolite, diacetylhydrazine [5]. The *NAT2* gene that is 870 bp long, positioned on chromosome 8 (8p22) short-arm, translates to the enzyme N-acetyltransferase 2 (290 amino acids long) [6]. *NAT2* gene basically codes for an important phase II enzyme that is basically expressed in more amount in the intestine and liver [7].

*NAT2* acts on different substrates. It is responsible for the catalyzation of the acetylation reaction of aromatic and heterocyclic amines. It particularly acts on arylhydrazine compounds present in medicines and thus helps in detoxification process. *NAT2* catalyzes the acetylation of INH, hydralazine, procainamide, sulphadoxine, dapsone and other clinically valuable drugs. Also, it catalyzes the acetylation of aromatic and heterocyclic carcinogens. *NAT2* is implicated in the modification of risk factors in the development of malignancies involving the urinary bladder, colorectal region, breast, prostate, lungs, and the head and neck region. Earlier studies have shown its involvement for the progress of the diseases like Alzheimer’s disease, schizophrenia, diabetes, cataract and parkinsonism [8–10].

The SNPs present in the coding region of a polymorphic *NAT2* gene can alter its enzymatic activity [11,12]. The variants found in *NAT2* gene severely affects the activity of INH, which results in three different phenotypes viz*.* fast acetylation phenotype, an intermediate and a slow acetylator phenotype. The three phenotypes differ in their alleles with the fast acetylation containing two alleles of wild-type, the intermediate containing one allele each of wild-type and mutant-type, and the slow acetylator containing two alleles of mutant-type.

The individuals with rapid acetylation capacity contain homozygous or heterozygous *NAT2* alleles (wild-type), and with slow acetylation contain homozygous mutant alleles [13]. The common SNPs [rs1799929 (481C>T), rs1799930 (590G>A) and rs1799931 (857G>A)] reported in *NAT2* gene are found in its coding region and these SNPs are accountable for significantly decreasing the acetylation capacity, which further slows down acetylator phenotype [11]. The biological significance of *NAT2* gene polymorphisms have made many scientists to peek thoroughly into the relationship of *NAT2* polymorphisms with INH-induced hepatotoxicity in TB patients among different populations [14–25]. But the previously published studies have demonstrated the inconsistent results. The inconsistency in their findings may be because of their small sample (cases/controls) size and weak statistical power. The scientists consider large sample sizes to be good to establish genetic associations with complex diseases [26]. It is well established that single studies may have been insufficient to detect the overall effects. As a solution, a meta-analysis is a very good dependable statistical tool to perform a quantitative analysis that can overcome the limitations of individual studies with small sample sizes (showing inadequate statistical power) and inconsistent results [27,28]. A meta-analysis combines multiple studies on the same alleles of genes to enhance the statistical power of the analysis and gives more accurate and reliable results of the genetic effects.

Therefore, we executed this meta-analysis by combining all the eligible published studies to examine the comprehensive picture of the above said pharmacogenetic association and understand the role of *NAT2* gene polymorphisms in INH-induced hepatotoxicity in TB patients. Moreover, the overall quality of the present study was evaluated by quality score analysis using Newcastle–Ottawa Scale (NOS). Additionally, the Trial Sequential Analysis (TSA) was carried out to reduce type I statistical errors like publication bias and random errors in order to estimate the statistical consistency of the quantitative data used in this cumulative meta-analysis, taking into account the threshold of statistical significance. Concisely, the current trial sequential meta-analysis of the published case–control reports will help in strengthening the postulated pharmcogenetic association of *NAT2* genetic variants with INH-induced hepatotoxicity risk in TB patients.

## Materials and methods

### Identification and eligibility of relevant studies

Various scholarly web-databases like Google Scholar, PubMed (Medline) and EMBASE were searched to find well-matched and peer reviewed research studies. The last search performed was updated on December 2017 using the following keyword combinations: N-acetyltransferase 2 OR *NAT2* OR gene (variant OR polymorphism OR mutation) AND tuberculosis induced hepatotoxicity OR TB induced hepatotoxicity. The focus of the search was human studies only. The articles recovered through search were evaluated by examining their titles and abstracts, and were also checked for their suitability according to the preset criteria for this meta-analysis. Furthermore, a manual search was conducted for the pertinent articles from the reference lists present in the retrieved articles to analyze the more useful articles, and to lessen the possibility of missing any relevant study from the keyword searches.

### Inclusion and exclusion criteria

The inclusion criteria considered the following points in the studies to be included: (a) must have evaluated the association between *NAT2* gene polymorphisms and INH-induced hepatotoxicity in TB patients, (b) must have used TB case(s) with INH-induced hepatotoxicity and TB control(s) without INH-induced hepatotoxicity in their study design, (c) must have genotype frequencies available for both cases and controls, (d) must have defined inclusion and exclusion criteria for the study subjects and clear definitions of TB- and INH-induced hepatotoxicity and e) must have been printed in English language. In addition to the above-mentioned criteria, if the data for the same patient population were published at more than one place, we included only the most recent or complete study in this meta-analysis.

**Figure 1:**
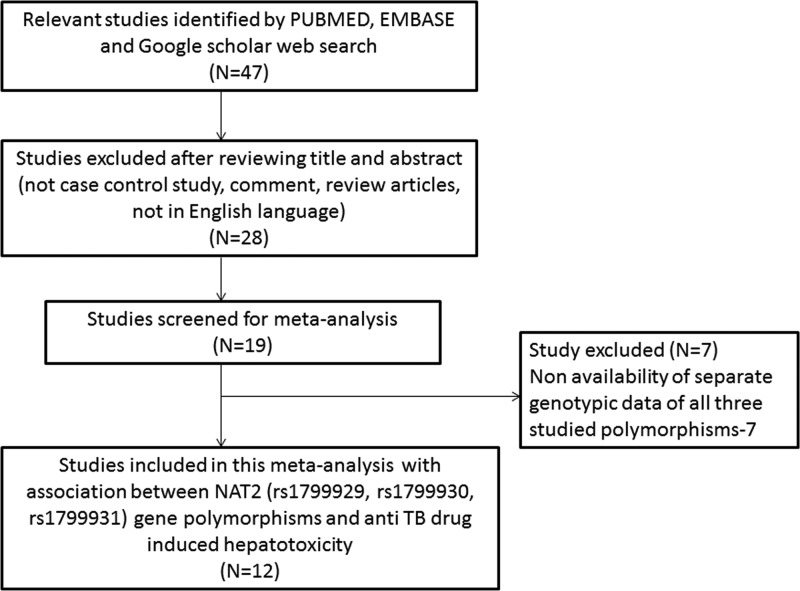
PRISMA 2009 Flow-diagram showing the identification and selection process (inclusion/exclusion) of the pertinent studies for the present meta-analysis

Studies reporting (a) TB patients with virus co-infection (HBV, HCV or HIV), (b) pure therapy without complete distribution of *NAT2* polymorphisms, (c) data overlapping, (d) involving cases only and (e) review articles were excluded from this meta-analysis. The information concerning the identification and selection (inclusion/exclusion) of the pertinent studies for the present meta-analysis is depicted in PRISMA 2009 Flow-diagram (Figure 1).

### Literature search strategy

Two researchers (S.K. and R.K.M.) independently examined all the titles and abstracts available from the retrieved publications using the selected online databases search in a chronological manner. The full-texts of all the publications deemed potentially qualified were retrieved. In order to assess the eligibility for the inclusion of the study, one investigator (S.K.) thoroughly appraised all the full-text articles. Following the previous step, the second investigator (R.K.M.) repeated the same evaluation process independently by a random selection of 10% of the full-text articles, and it was found that there was a full agreement between the two researchers regarding the selection criteria and study exclusion. Subsequently, for identification of the final set of the eligible articles, one investigator (S.K.) extrapolated the relevant data from all the studies. To cross-check the above step, another investigator (R.K.M.) independently reappraised the collected information from all the included studies. Any discrepancy associated with the study selection was resolved by a comprehensive discussion with the third investigator (S.H.), contributed as arbitrator.

### Screening of the studies

The electronic searches performed on selected web-databases resulted 47 research articles in the initial stage. But, after many rounds of screening by applying above stated preset selection (inclusion/exclusion) criteria only 12 studies were found eligible to be included here in this analysis as they dealt with *NAT2* 481C>T, 590G>A, 857G>A gene polymorphisms and INH-induced hepatotoxicity in TB patients. The full texts of the 12 selected articles were scanned thoroughly for relevance to the present study. Survival studies with *NAT2* polymorphic patients having INH-induced hepatotoxicity were immediately excluded. Also excluded were the expression studies probing *NAT2* at mRNA or protein level. Review articles were also disqualified from inclusion in the present study. Only studies showing all genotypic frequencies and having a case–control/cohort design were considered for inclusion in the present study. Scrutinizing carefully the reference lists of the selected articles, to find other articles relevant to this study, revealed no pertinent studies fitting the criteria adopted.

### Quality assessment by Newcastle–Ottawa Scale criteria

The established NOS criteria of quality score analysis was adopted for the methodological quality assessment of the included studies (i.e. *n*=12) [29]. The quality assessment using NOS criteria was done separately by two independent researchers. It included three aspects: (a) selection of the subjects: 0–4 points; (b) subject comparability: 0–2 points; (3) clinical outcome: 0–3 points. Studies scoring 5 or more stars were considered to be of moderate to high quality [30]. The quality evaluation of the retrieved studies was done by two investigators (S.A.D. and R.K.M.), independently, and the scoring inconformity (if any) was resolved by thorough discussion in the presence of a third investigator (participated as adjudicator: S.H.).

### Data extraction

The methodical standard data extraction procedure was used for each recovered publication. The publications were reviewed independently by two investigators (S.K. and R.K.M.). Standard data-collection form was used to make sure that the collected information was correct. It was guaranteed by strictly applying the inclusion/exclusion criteria as stated earlier. The main characteristics curtailed from the recovered studies encompassed, author’s first name, publication year, size of cases and controls, the country of origin, study type, association/no association or lack of association status, genotyping methods and frequencies for cases and controls. Any type of discrepancy or disagreement on any data item, summarized from the collected studies, between the two investigators (i.e. S.K. and R.K.M.) were resolved by open scientific discussion in the presence of adjudicator (S.H.) in order to attain an absolute agreement.

### Publication bias diagnosis and heterogeneity evaluation

The publication bias present in the included studies was checked by funnel plot asymmetry and Egger’s regression statistics. A *P*-value of less than 0.05 was fixed for considering the significant publication bias. In additional, all the *NAT2* gene polymorphism related studies included in this meta-analysis were checked for heterogeneity using *Q*-test and *I*^2^ statistics.

### Statistical analysis

The level of intensity of the association between the studied SNPs and susceptibility to INH-induced hepatotoxicity was appraised by computing crude ORs and their corresponding 95% CI. The combined ORs were estimated for allele contrast, log-additive, dominant and recessive models [31]. The chi-square-based *Q*-test was used to test the heterogeneity assumption between the studies [32], which was considered statistically significant when *P*-value was less than 0.05. This was done to avoid the underestimation of heterogeneity presence. The fixed-effect model was used for the pooling the results when *P*-value was greater than 0.05 [33], otherwise, the random effects model was applied [34]. Furthermore, *I*^2^ statistics was used to efficiently test the heterogeneity [35]. The chi-squared test was used to compute the Hardy–Weinberg equilibrium (HWE) for the control population. The funnel plot asymmetry was measured by applying Egger’s linear regression test. The Egger’s linear regression test uses linear regression approach to compute the funnel plot asymmetry on the natural logarithmic scale of the ORs. The *t*-test was applied to estimate the significance of the intercept, wherein the *P*-value of less than 0.05 denoted statistically significant publication bias [36,37]. The Comprehensive Meta-Analysis (CMA) V2 software program (Biostat, U.S.A.) was used to perform all the statistical calculations stated above. The *P*-values were two-sided and the threshold of significance was *P*<0.05.

### Trial sequential analysis

As per the Cochrane handbook, meta-analyses studies are thought to be best when the analysis include all the eligible trials. However, many a times it cannot be considered as adequate evidence, because during the meta-analysis systematic errors (bias) or random errors (play of chance) may occur. The errors have been minimized by the use of a novel statistical analysis software, namely, TSA tool available from Copenhagen Trial Unit, Center for Clinical Intervention Research, Denmark. TSA helps in estimation of the required information size and also in adjustment of the threshold to achieve statistical significance that leads to measurement of the power of analysis conclusion [38–41]. A robust evidence is confirmed when the TSA monitoring boundary is crossed by the *Z* curve before reaching the required information size, and then subsequent trials are not required. However, if the *Z* curve fails to cross the monitoring boundaries before the required information size is reached, continuing of trials becomes necessary. We used Trial Sequential Analysis software program version 0.9 (http://www.ctu.dk/tsa/) for analysis in the present study.

## Results

### Characteristics and quality assessment of the studies

Tables 1 and 2 present the key characteristics, and genotype distribution along with minor allele frequency (MAF) of the cases and controls of all the 12 included studies in this meta-analysis. All the studies were evaluated for their quality according to the scoring system of NOS and majority of the studies (approximately 80%) were found to score 5 stars or more. This indicates a moderate to good quality of all the included studies in this pooled analysis (Supplementary Table S1). The chronological strategy for selecting the relevant studies used in this meta-analysis is given as PRISMA 2009 Flow Diagram (Figure 1).

**Table 1:** Main characteristics of all the 12 studies included in the present meta-analysis

First author and year	Country	Ethnicity	Type of Study	Controls	Cases	*NAT2* gene polymorphisms	Methods	Drug therapy	Association
Yuliwulandari et al., 2016	Indonesia	Asian	HB	191	50	481C>T 590G>A 857G>A	Sequencing	INH+RMP+PZA	590G>A
Xiang et al., 2014	China	Asian	HB	1858	386	481C>T 590G>A 857G>A	Other sources	INH+RMP+PZA+EMB	481C>T
Singh et al., 2014	India	Asian	HB	135	50	481C>T 590G>A	Sequencing	INH+RMP+PZA	481C>T 590G>A
Santos et al., 2013	Brazil	Mixed	HB	252	18	481C>T 590G>A 857G>A	Sequencing	INH+RMP+PZA	No
Gupta et al., 2013	India	Asian	HB	165	50	481C>T 590G>A	PCR-RFLP	INH+RMP+PZA	No
Mishra et al., 2013	India	Asian	HB	173	33	481C>T 590G>A 857G>A	PCR-RFLP	INH+RMP+PZA+EMB	590G>A
Ben Mahmoud et al., 2012	Tunisia	African	HB	52	14	481C>T 590G>A 857G>A	PCR-RFLP	INH+RMP	481C>T 590Ggt;A
An et al., 2012	China	Asian	HB	107	101	481C>T 590G>A 857G>A	Sequencing	INH+RMP+PZA+EMB	857G>A
Lv et al., 2012	China	Asian	HB	356	89	481C>T 590G>A 857G>A	PCR-RFLP	INH+RMP+PZA+EMB	No
Lee et al., 2010	Taiwan	Asian	HB	95	45	481C>T 590G>A 857G>A	Taq Man	INH+RMP+PZA	857G>A
Kim et al., 2009	Korea	Asian	HB	159	67	590G>A 857G>A	Tagging-SNP	INH+RMP+PZA+EMB	590G>A
Bozok et al., 2008	Turkey	Caucasian	HB	70	30	481C>T 590G>A 857G>A	Mutation detection kit	INH+RMP+PZA+EMB	590G>A

Abbreviations: EMB, ethambutol, HB, Hospital based, INH, isoniazid; PZA, pyrazinamide; RMP, rifampicin.

**Table 2:** Genotypic distribution of *NAT2* gene polymorphisms included in this meta-analysis

First author and year	Controls				Cases				HWE
	Genotype			Minor allele	Genotype			Minor allele	
**481C>T (rs1799929)**	**CC**	**CT**	**TT**	**MAF**	**CC**	**CT**	**TT**	**MAF**	***P*****-value**
Yuliwulandari et al., 2016	146	42	3	0.125	38	11	1	0.130	0.991
Xiang et al., 2014	928	397	62	0.187	197	111	9	0.203	0.021
Singh et al., 2014	62	63	10	0.307	14	26	10	0.460	0.264
Santos et al., 2013	96	120	36	0.380	9	5	4	0.361	0.878
Gupta et al., 2013	86	68	11	0.272	16	23	11	0.450	0.617
Mishra et al., 2013	79	78	16	0.317	15	18	0	0.272	0.602
Ben Mahmoud et al., 2012	24	18	10	0.365	4	4	6	0.571	0.067
An et al., 2012	103	4	0	0.018	95	6	0	0.029	0.843
LV et al., 2012	333	23	0	0.032	81	8	0	0.044	0.528
Lee et al., 2010	3	8	84	0.926	0	5	40	0.944	0.001
Bozok et al., 2008	39	24	7	0.271	12	16	2	0.333	0.265
**590G>A (rs1799930)**	**GG**	**GA**	**AA**	**MAF**	**GG**	**GA**	**AA**	**MAF**	***P*-value**
Yuliwulandari et al., 2016	84	89	18	0.327	17	21	12	0.450	0.420
Xiang et al., 2014	801	465	114	0.251	159	118	27	0.282	0.001
Singh et al., 2014	38	44	22	0.423	14	25	3	0.369	0.173
Santos et al., 2013	241	6	5	0.031	17	1	0	0.027	0.001
Gupta et al., 2013	72	65	28	0.366	20	25	5	0.350	0.051
Mishra et al., 2013	93	62	18	0.283	7	21	5	0.469	0.122
Ben Mahmoud et al., 2012	26	24	2	0.269	7	5	2	0.321	0.212
An et al., 2012	72	32	3	0.177	54	35	12	0.292	0.804
LV et al., 2012	194	135	27	0.223	51	31	7	0.252	0.602
Lee et al., 2010	58	29	8	0.236	24	14	7	0.311	0.129
Kim et al., 2009	102	43	5	0.176	31	26	8	0.323	0.858
Bozok et al., 2008	41	26	3	0.45	9	15	6	0.228	0.655
**857G>A (rs1799931)**	**GG**	**GA**	**AA**	**MAF**	**GG**	**GA**	**AA**	**MAF**	***P*-value**
Yuliwulandari et al., 2016	138	52	1	0.141	32	17	1	0.190	0.093
Xiang et al., 2014	1103	274	20	0.112	256	57	1	0.093	0.527
Santos et al., 2013	210	40	2	0.087	12	5	1	0.194	0.949
Mishra et al., 2013	152	19	2	0.066	25	8	0	0.121	0.130
Ben Mahmoud et al., 2012	50	2	0	0.019	14	0	0	0	0.887
An et al., 2012	74	27	6	0.182	53	43	5	0.262	0.112
LV et al., 2012	268	80	8	0.134	66	22	1	0.134	0.487
Lee et al., 2010	68	25	1	0.143	28	9	8	0.277	0.431
Kim et al., 2009	116	34	2	0.125	49	16	1	0.136	0.901
Bozok et al., 2008	65	4	1	0.042	24	5	1	0.116	0.011

### Publication bias analysis

Publication bias was absent in all the genetic models as indicated by the funnel plots and the Egger’s test for *NAT2* 481C>T (Supplementary Table S2 and Supplementary Figure S1), 590G>A (Supplementary Table S3 and Supplementary Figure S2), 857G>A (Supplementary Table S4 and Supplementary Figure S3) gene polymorphisms.

### Heterogeneity assessment

In case of *NAT2* 481C>T polymorphism, two genetic models showed heterogeneity (Supplementary Table S2). Likewise, in case of *NAT2* 590G>A (Supplementary Table S3) and 857G>A (Supplementary Table S4) gene polymorphisms, three and one genetic models, respectively, depicted heterogeneity. Therefore, to generate the data for each polymorphism, we applied the random-effects model for the analysis.

### Quantitative synthesis

#### Association of NAT2 481C>T (rs1799929) gene polymorphism with INH-induced hepatotoxicity

**Figure 2:**
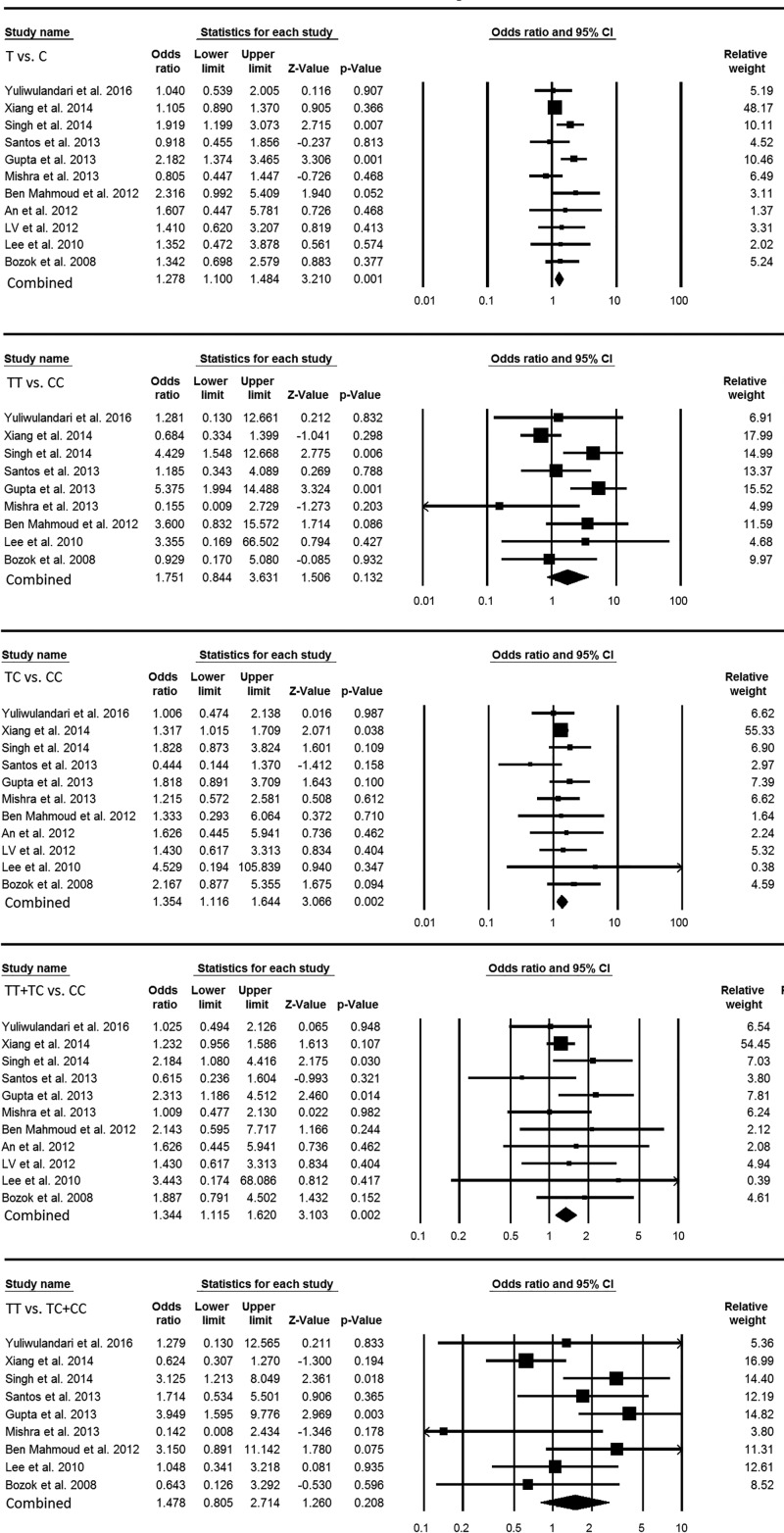
Forest plot of ORs with 95% CI of INH-induced hepatotoxicity risk associated with the *NAT2* 481C>T gene polymorphism for the overall population Note: Black square represents the value of OR and the size of the square indicates the inverse proportion relative to its variance. Horizontal line is the 95% CI of OR.

The 11 case–control studies depicting *NAT2* 481C>T polymorphism, comprising 3454 controls and 866 cases, provided enough data to calculate ORs. The *NAT2* 481C>T gene polymorphism demonstrated statistically significant association with INH-induced hepatotoxicity in allelic contrast (T vs. C: *P* = 0.001; OR = 1.278, 95% CI = 1.100–1.484), heterozygous (TC vs. CC: *P* = 0.002; OR = 1.354, 95% CI = 1.116–1.644) and dominant (TT+TC vs. CC: *P* = 0.002; OR = 1.344, 95% CI = 1.115–1.620) genetic models as revealed by the pooled ORs. On the contrary, homozygous (TT vs. CC: *P* = 0.132; OR = 1.751, 95% CI = 0.844–3.631) and recessive (TT vs. TC+CC: *P* = 0.208; OR = 1.478, 95% CI = 0.805–2.714) genetic models failed to demonstrate any significant association (Figure 2).

#### Association of NAT2 590G>A (rs1799930) gene polymorphism with INH-induced hepatotoxicity

**Figure 3:**
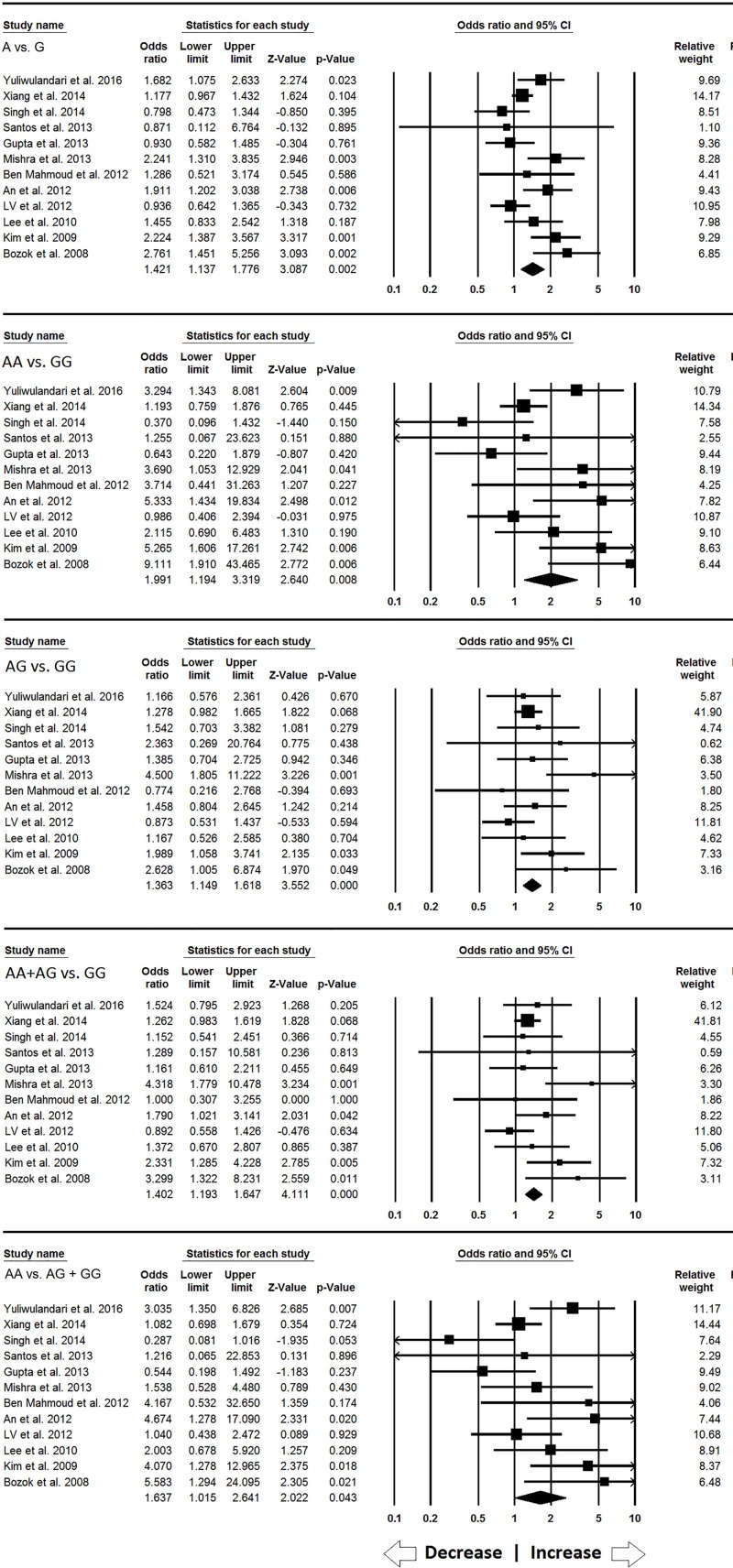
Forest plot of ORs with 95% CI of INH-induced hepatotoxicity risk associated with the *NAT2* 590G>A gene polymorphism for the overall population Note: Black square represents the value of OR and the size of the square indicates the inverse proportion relative to its variance. Horizontal line is the 95% CI of OR.

For *NAT2* 590G>A, 12 case–control studies containing 3613 controls and 933 cases provided enough data to calculate ORs. The pooled ORs showed that allelic (A vs. G: *P* = 0.002; OR = 1.421, 95% CI = 1.137–1.776), homozygous (AA vs. GG: *P* = 0.008; OR = 1.991, 95% CI = 1.194–3.319), heterozygous (AG vs. GG: *P* = 0.001; OR = 1.363, 95% CI = 1.149–1.618), dominant (AA+AG vs. GG: *P* = 0.001; OR = 1.402, 95% CI = 1.193–1.647) and recessive (AA vs. AG+GG: *P* = 0.043; OR = 1.637, 95% CI = 1.015–2.641) genetic models demonstrated increased risk of INH-induced hepatotoxicity as compared with the wild-type (Figure 3).

#### Association of NAT2 857G>A (rs1799931) gene polymorphism with INH-induced hepatotoxicity

**Figure 4:**
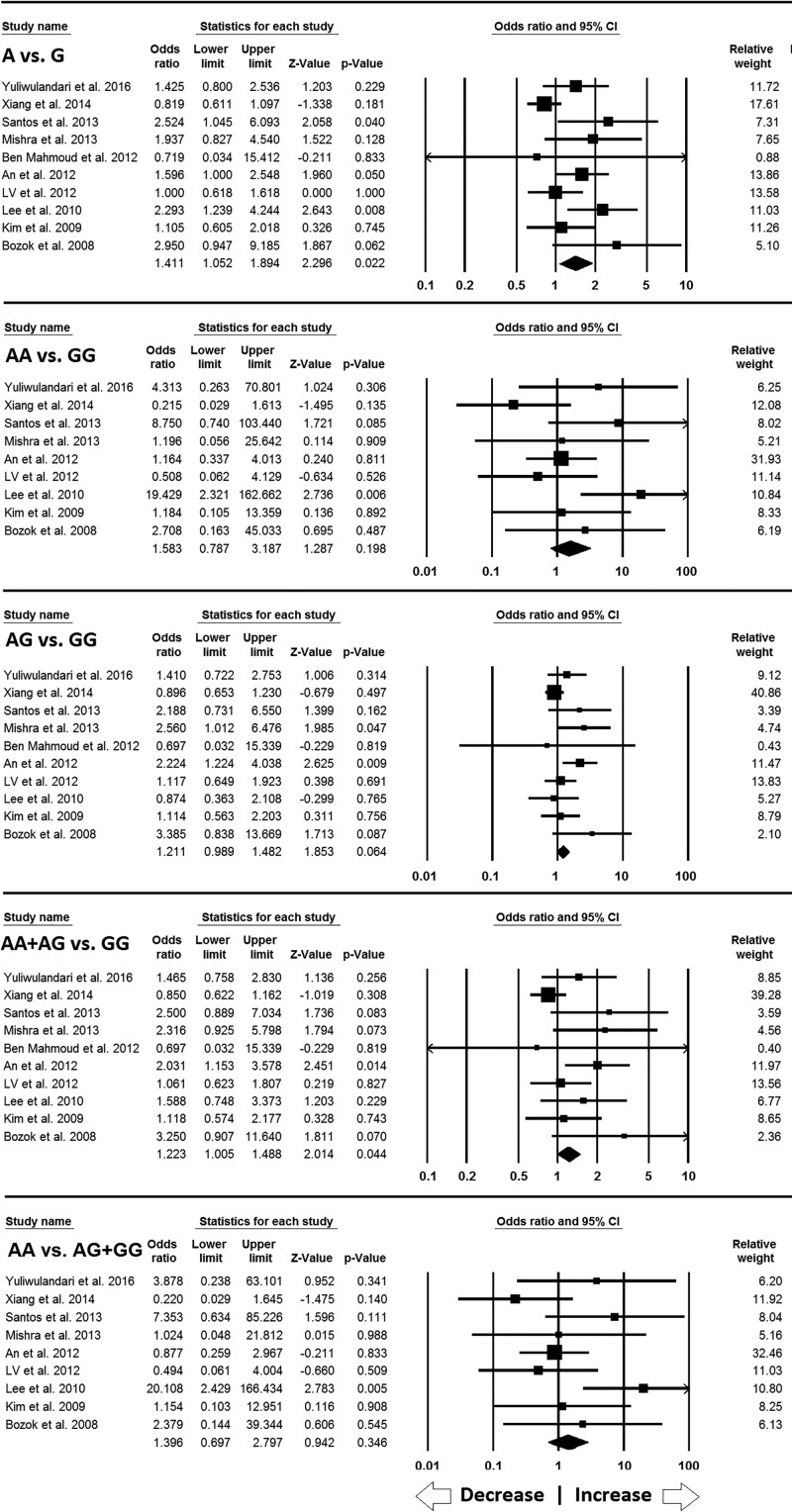
Forest plot of ORs with 95% CI of INH-induced hepatotoxicity risk associated with the *NAT2* 857G>A gene polymorphism for the overall population Note: Black square represents the value of OR and the size of the square indicates the inverse proportion relative to its variance. Horizontal line is the 95% CI of OR.

A total of 10 case–control studies for *NAT2* 857G>A polymorphism, including 3313 controls and 833 cases, provided enough data to calculate ORs. The allelic (A vs. G: *P* = 0.022; OR = 1.411, 95% CI = 1.052–1.894) and dominant (AA+AG vs. GG: *P* = 0.044; OR = 1.223, 95% CI = 1.005–1.488) genetic model showed positive association with INH-induced hepatotoxicity, as shown by pooled ORs. Whereas homozygous (AA vs. GG: *P* = 0.198; OR = 1.583, 95% CI = 0.787–3.187), heterozygous (AG vs. GG: *P* = 0.064; OR = 1.211, 95% CI = 0.989–1.482) and recessive (AA vs. AG+GG: *P* = 0.346; OR = 1.396, 95% CI = 0.697–2.797) genetic models did not reveal any risk with INH-induced hepatotoxicity (Figure 4).

### Sensitivity analysis

To assess the individual study influence on the overall risk of INH-induced hepatotoxicity, the leave-one-out sensitivity analysis was performed and pooled ORs were recomputed for each *NAT2* gene polymorphism. The recomputed ORs for each polymorphism were indifferent from the primary values [481C>T (Supplementary Figure S4), 590G>A (Supplementary Figure S5), 857G>A (Supplementary Figure S6)] that guaranteed the stability of the overall results.

### Trial sequence analysis

**Figure 5:**
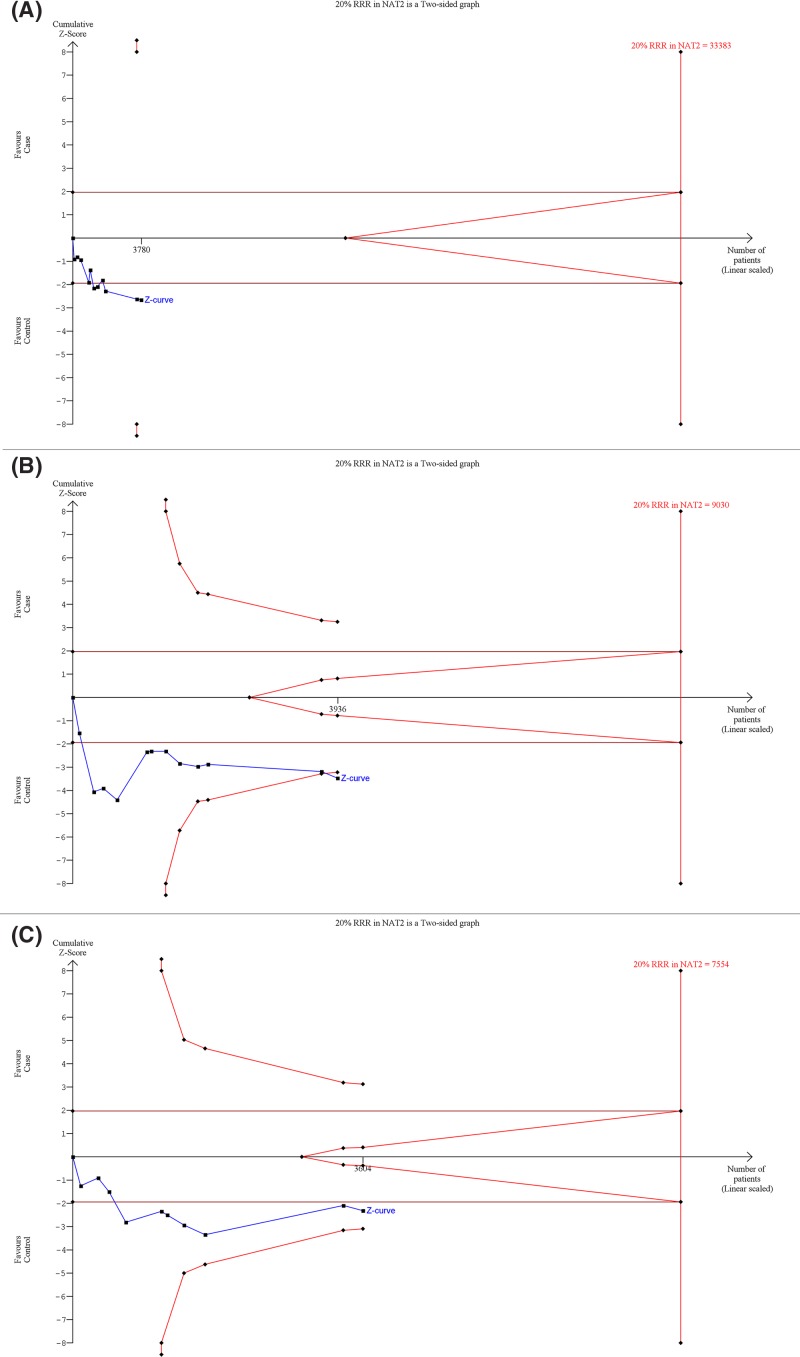
Trial sequence analysis of all the included studies dealing with *NAT2* gene polymorphisms based on dominant genetic model: (A) 481C>T, (B) 590G>A, (C) 857G>A and INH-induced hepatotoxicity risk

TSA was applied to investigate the precise association between *NAT2* 481C>T, 590G>A, 857G>A gene polymorphisms and INH-induced hepatotoxicity risk by minimizing the random errors. Using the dominant model as an example for all the three studied polymorphisms of *NAT2* gene, the TSA was performed. The TSA results indicate insufficiency of cumulative evidence advising further trials (Figure 5A,B,C). Before reaching the desired power, the cumulative *Z* curve was unable to cross the trial monitoring boundary. Other models also depicted similar findings (data not shown).

## Discussion

Many efforts have been done in the past decades to bring the TB disease under control, but most of them proved to be heart-wrenching. The risk for progression of anti-tuberculosis drug induced hepatotoxicity affecting humans is a serious and one of the most frequent side effects [42]. It poses a major threat to both the clinician and the patients and is considered as the key reason for discontinuation of anti-tuberculosis drugs and poorer outcome of TB. Although a large number of patients are exposed to anti-tuberculosis drugs across the world, but the pathogenesis underlying hepatotoxicity has not been fully elucidated till date. The most consistent association of hepatotoxicity is related to host factors that may influence the risk of an individual to develop such hepatotoxicity. Lately, the genetic factor encoding metabolic enzymes have been identified as risk factors for hepatotoxicity, and the focus of research has been shifted to anti-tuberculosis drug therapy to predict hepatotoxicity and reduce its incidence [43]. Hepatotoxicity of INH treatment derives from INH itself (a hydrazine derivative). The metabolites, INH like acetyl-hydrazine, hydrazine and ammonia are considered to be involve in the formation of reactive oxygen species that can cause necrosis and autoimmunity [44]. They are also thought to be involved in epigenetic effects [45]. The slow and rapid acetylated phenotypes of INH were reported about nearly 60 years ago in TB patients [46]. This difference was shown to be possibly due to genetic variability of *NAT2* enzyme that mediates the bioconversion of INH to its metabolite acetyl INH. Acetyl INH is hydrolyzed to acetyl hydrazine and further acetylated by *NAT2* to non-toxic diacetyl hydrazine. In case of low *NAT2* activity, acetyl hydrazine is predominantly oxidized by CYP2E1 leading to increased hepatotoxicity [47]. Hydrazine is the major hepatotoxic metabolite of INH and its quantity is related to the acetylation rate of *NAT2* [48]. Hepatocytes can be destroyed by anti-TB drug metabolites. Reactive metabolites bind to plasma proteins of hepatocytes that act as haptens, interfering in cell homeostasis or eliciting immunological response [49]. Single-nucleotide polymorphisms 481C>T, 590G>A and 857G>A within the *NAT2* gene can affect the function of *NAT2* enzyme resulting in reduced stability, altered affinity for the substrate, or a protein that is targeted for proteosome degradation [50].

Considering the pharmacogenetic significance of *NAT2* gene in view, previously several studies [14–25] have tried to establish a possible link between *NAT2* 481C>T, 590G>A, and 857G>A gene variants and the development of INH-induced hepatotoxicity in TB patients from different populations but failed to give concrete conclusion possibly due to small size, and still the said relationship is controversial. Generally, small sample sizes makes studies statistically weak, thereby, less likely to detect small risk factors of the disease. A well-organized meta-analysis investigating the relationship between *NAT2* 481C>T, 590G>A, and 857G>A gene polymorphisms and risk of developing INH-induced hepatotoxicity in TB patients is still wanting. Therefore, we conducted this trial sequential meta-analysis to precisely investigate the pharmacogenetic contribution of these three polymorphisms of *NAT2* gene in overall INH-induced hepatotoxicity risk. On the basis of 12 case–control design based independent publications, we in this meta-analysis proved with the help of statistical data that individuals carrying variant allele of 481C>T, 590G>A and 857G>A polymorphisms of *NAT2* gene had higher risk of INH-induced hepatotoxicity than those with the wild allele of these polymorphisms. It supports the significant role of *NAT2* gene polymorphisms in the etiology of hepatotoxicity. The *NAT2* gene polymorphisms reduce the metabolism of INH in the liver, which is linked to an increased hepatotoxicity risk. Previously published phyenotypic studies also reported that all the anti-tuberculosis drugs, i.e. INH, RMP, PZA and EMB, usually induce hepatotoxicity and may follow different pathogenic mechanisms [51,52]. In combination therapy, the main population of *M. tuberculosis* is susceptible to INH; however, PZA is effective against slow growing bacilli in acidic milieus, and RMP against non-replicating bacilli [53]. *NAT2* gene plays a first key role in hepatotoxicity caused by INH and RMP combination therapies, and RMP is known to reduce *NAT2* activity and may act as a potent inducer of CYP2E1 [54]. An *in vitro* study has also shown that INH and its toxic metabolite, hydrazine, increases the toxicity of PZA to hepatocytes [55]. In this manner, *NAT2* is indirectly involved to affect the metabolism of other ATD drugs except INH.

Nowadays, the pharmacogenetic testing has been more and more applied to identify and exclude the individuals with a certain genetic makeup for the treatment in the clinical practice. Hence, *NAT2* genotyping is warranted before the starting of the administration of anti-tuberculosis drug therapy, this will ultimately help the clinicians to personalize and optimize the treatments régimes, making them therapeutically more efficient while simultaneously minimizing the side effects associated with the use of these drugs.

In addition to the precise conclusion, some advantageous attributes of the present trial sequence meta-analysis are: first, the quality wise evaluation of the chosen 12 studies was sternly done by following NOS quality scoring, wherein most of the studies attained five or more stars suggesting each individual study as of modest to good quality. The NOS quality analysis took into account the sample size, inclusion criteria for TB patients with INH-induced hepatotoxicity, genotypes and inclusion of TB patients without hepatotoxicity as controls for each study. Second, strict and explicit search and preset selection procedure was adopted for the inclusion of the studies. Third, the procedural issues, e.g. publication bias and sensitivity, usually arising in meta-analysis were well explored (funnel plot asymmetry indicated no apparent publication bias that adds to the credibility and statistical robustness of our results), which further confirmed the validity and reliability of the present study. The outcome of the leave-one-out sensitivity analysis also depicted no significant effect on cumulative ORs and their corresponding CIs. Fourth, TSA was also performed for strengthening the conclusion and reducing the type I error rate.

It is well established that interim analyses increase the risk of type I error in a single trial [53,54]. However, recent reports stated that spurious conclusions in traditional or conventional or cumulative meta-analyses can be reduced by using TSA because it combines the available studies by time series, which otherwise have chances of producing random errors due to repeat testing on amassing data. Various empirical studies have showed that the TSA gives better control of random errors than the traditional naïve meta-analysis [38]. Applying TSA with meta-analysis uses transparent assumptions and gives better control of type I and II errors than meta-analysis alone where naïve unadjusted CIs are used [38]. TSA minimizes the random errors by calculating the number of subjects or information size required to associate or dissociate a certain effect during the meta-analysis. The plausible relative risk reduction of 10 to 30% or that observed in low bias risk trials usually forms the basis of the information size. The information size along with type I and II error risk helps in construction of the threshold for achieving statistical significance. This means that TSA sets the required sample size and boundaries for the meta-analysis to determine reliability and conclusiveness of the evidence [39].

The potential limitations of the current pooled study were: (a) heterogeneity was detected in few genetic models, so random-effects model was applied to get wider CIs, (b) the studies published in English were included for the current meta-analysis, it is quite obvious that some relevant studies might published in other languages may have overlooked, (c) only most famous web-databases were searched, it is possible that some pertinent studies available in other databases may have skipped and (d) only three SNPs were considered for the current meta-analysis because of their wide reporting in independent studies; this might have led to the possibility of oversighting other SNPs affecting INH metabolism.

In summary, our meta-analysis supports that TB affected individuals with *NAT2* 481C>T, 590G>A and 857G>A gene polymorphisms have increased risk of developing INH-induced hepatotoxicity. These results will help in improving the understanding related to the role of *NAT2* 481C>T, 590G>A and 857G>A gene polymorphisms and assist in identifying the individuals prone to disease, which could be helpful in achieving optimal treatment of individual TB patients. Further to this, the present study warrants to perform the additional studies with large sample size to investigate the potential molecular mechanism underlying INH-induced hepatotoxicity and facilitate the development of molecular approaches to detect the susceptible genotype, and to verify the current findings reported in this manuscript.

## Supporting information

**Figure SI1:** 

**Figure SI2:** 

**Figure SI3:** 

**Figure SI4:** 

**Figure SI5:** 

**Figure SI6:** 

**Table SI1:** Quality assessment according to the Newcastle-Ottawa Scale for all the studies included in the present metaanalysis

**Table SI2:** Statistics to test publication bias and heterogeneity in the present meta-analysis for *NAT2* 481C>T gene polymorphism and ATD induced hepatotoxicity

**Table SI3:** Statistics to test publication bias and heterogeneity in the present meta-analysis for *NAT2* 590G>A gene polymorphism and ATD induced hepatotoxicity

**Table SI4:** Statistics to test publication bias and heterogeneity in the present meta-analysis for *NAT2* 857G>A gene polymorphism and ATD induced hepatotoxicity

## Abbreviations

INH: isoniazid or isonicotinylhydrazide
MAF: minor allele frequency
*NAT2*: N-acetyltransferase 2
NOS: Newcastle–Ottawa Scale
PZA: pyrazinamide
RMP: rifampicin
TB: tuberculosis
TSA: Trial Sequential Analysis
