# Supplementary material for: Pharmacogenetic association between NAT2 gene polymorphisms and isoniazid induced hepatotoxicity: trial sequence meta-analysis as evidence
Source: Biosci Rep. 2019 Jan 15;39(1):BSR20180845. doi: 10.1042/BSR20180845 (PMC6331676; doi:10.1042/BSR20180845)
Supplement: Supplementary file 1 [file bsr20180845_Supp1.pdf]

SUPPLEMENTARY INFORMATION

Pharmacogenetic Association Between NAT2 Gene Polymorphisms and Isoniazid Induced Hepatotoxicity: Trial Sequence Meta-analysis as Evidence

Table SI1. Quality assessment according to the Newcastle-Ottawa Scale for all the studies included in the present meta-analysis

First author and year	Quality indicators		
	Selection	Comparability	Exposure
Yuliwulandari et al., 2016	**	*	**
Xiang et al., 2014	**	*	**
Singh et al., 2014	**	*	***
Santos et al., 2013	**	*	**
Gupta et al., 2013	**	*	**
Mishra et al., 2013	***	*	**
Xiaozhen et al., 2012	***	*	***
Mahmoud et al., 2012	**	*	**
An et al., 2012	**	*	**
Lee et al., 2010	**	*	**
Kim et al., 2009	**	*	**
Bozok et al., 2008	**	*	**

Note: On assessing the quality of all the included studies by using the *Newcastle-Ottawa Scale*, all the studies scored five stars or more, indicating no bias.

Table SI2. Statistics to test publication bias and heterogeneity in the present meta-analysis for *NAT2* 481C>T gene polymorphism and Isoniazid induced hepatotoxicty

Comparisons	Egger's regression analysis			Heterogeneity analysis			Model used for the present meta-analysis
	Intercept	95% Confidence Interval	p-value	Q-value	$P_{heterogeneity}$	I^2 (%)	
T vs C	0.65	-1.08 to 2.38	0.42	15.47	0.12	35.37	Fixed
TT vs CC	0.07	-3.09 to 3.22	0.96	19.33	0.01	58.62	Random
TC vs CC	0.16	-0.94 to 1.26	0.75	7.46	0.68	0.001	Fixed
TT+TC vs CC	0.52	-0.73 to 1.77	0.37	10.05	0.44	0.530	Fixed
TT vs TC+CC	-0.55	-4.09 to 2.98	0.72	18.00	0.02	55.56	Random

Table SI3. Statistics to test publication bias and heterogeneity in the present meta-analysis for *NAT2* 590G>A gene polymorphism and Isoniazid induced hepatotoxicty

Comparisons	Egger's regression analysis			Heterogeneity analysis			Model used for the present meta-analysis
	Intercept	95% Confidence Interval	p-value	Q-value	$P_{heterogeneity}$	I^2 (%)	
A vs G	0.99	-1.28 to 3.25	0.35	27.56	0.004	60.09	Random
AA vs GG	1.29	-0.89 to 3.47	0.22	26.89	0.005	59.09	Random
AG vs GG	0.83	-0.70 to 2.37	0.25	14.52	0.210	24.23	Fixed
AA+AG vs GG	0.97	-0.74 to 2.68	0.24	18.31	0.070	39.92	Fixed
AA vs AG+GG	1.07	-1.18 to 3.32	0.31	26.21	0.006	58.03	Random

Table SI4. Statistics to test publication bias and heterogeneity in the present meta-analysis for *NAT2* 857G>A gene polymorphism and Isoniazid induced hepatotoxicty

Comparisons	Egger's regression analysis			Heterogeneity analysis			Model used for the present meta-analysis
	Intercept	95% Confidence Interval	p-value	Q-value	$P_{heterogeneity}$	I^2 (%)	
A vs G	2.01	0.02 to 4.00	0.05	19.64	0.02	54.19	Random
AA vs GG	1.14	-2.48 to 4.76	0.48	13.05	0.11	36.69	Fixed
AG vs GG	1.38	-0.34 to 3.11	0.10	14.16	0.12	36.43	Fixed
AA+AG vs GG	1.80	0.14 to 3.46	0.04	15.45	0.08	41.74	Fixed
AA vs AG+GG	1.33	-2.21 to 4.88	0.40	13.35	0.10	40.07	Fixed

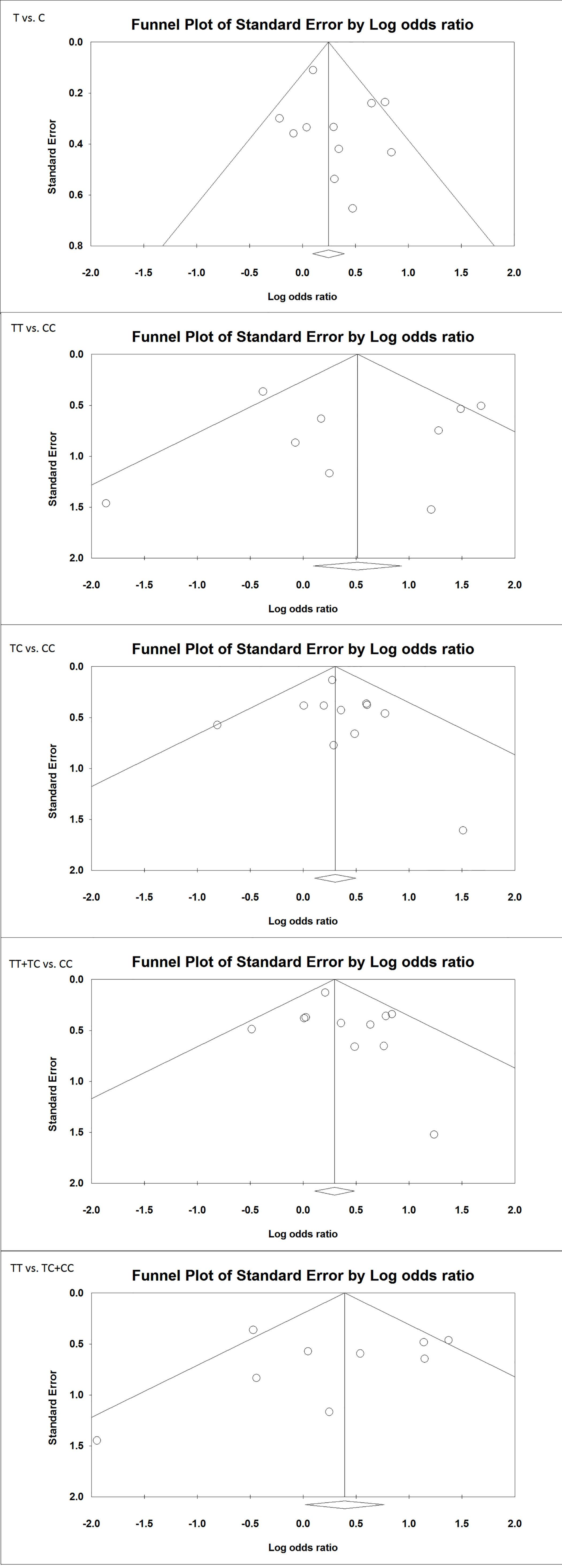

Figure SI1: Assessment of publication bias shown with Funnel plots in studies assaying odds of INH induced hepatotoxicity with the NAT2 481C>T (rs1799929) gene polymorphism for overall analysis (Odds ratio against standard error in different genetic models).

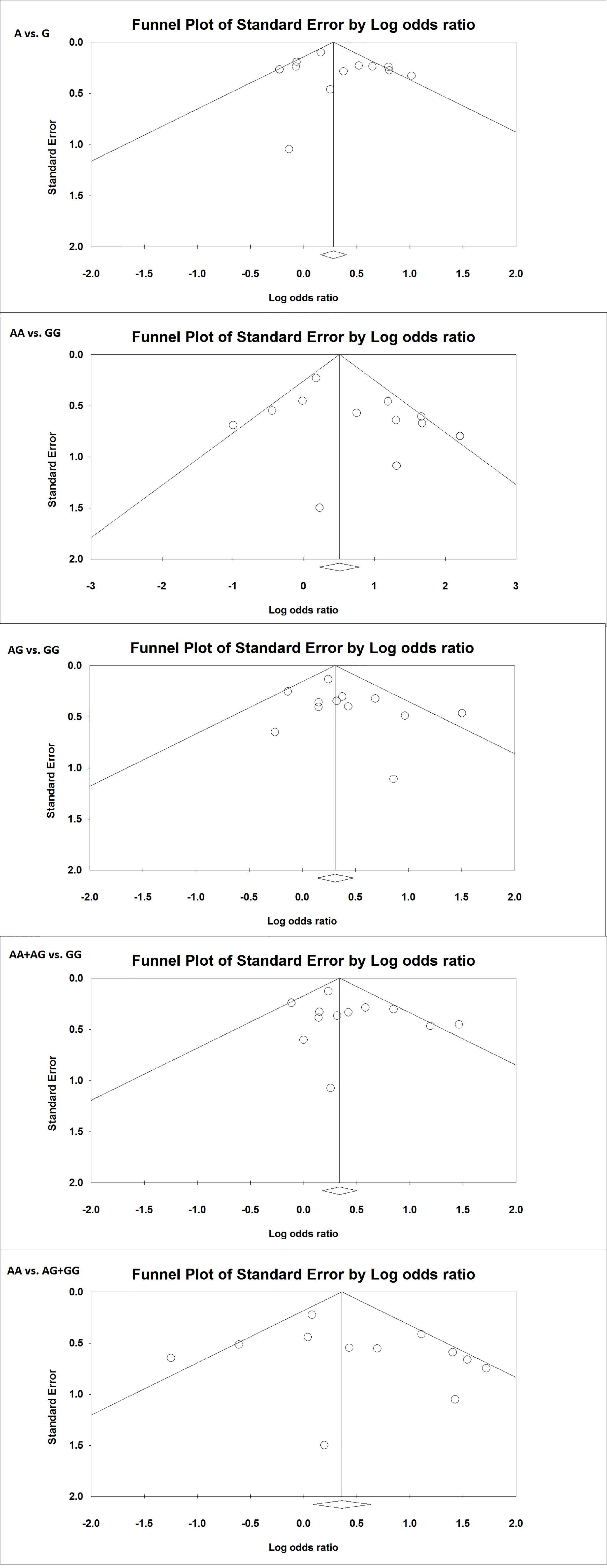

Figure SI2: Assessment of publication bias shown with Funnel plots in studies assaying odds of INH induced hepatotoxicity with the NAT2 590G>A (rs1799930) gene polymorphism for overall analysis (Odds ratio against standard error in different genetic models).

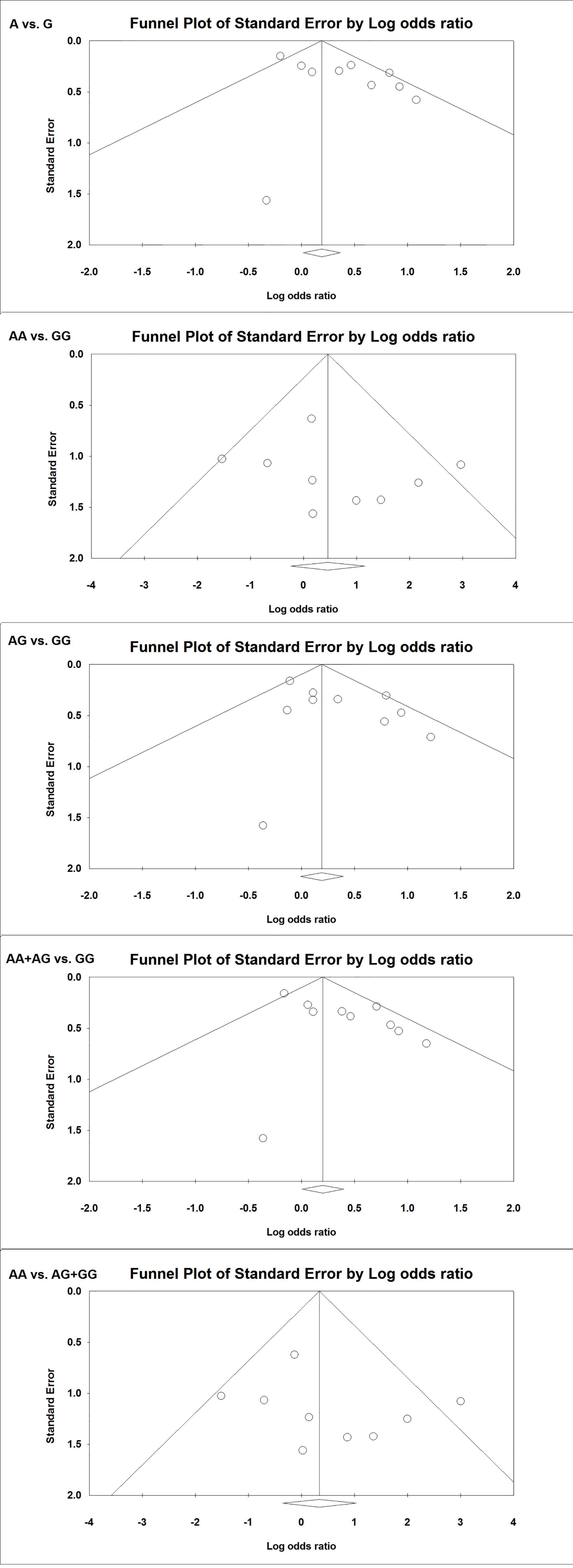

Figure SI3: Assessment of publication bias shown with Funnel plots in studies assaying odds of INH induced hepatotoxicity with the NAT2 857G>A (rs1799931) gene polymorphism for overall analysis (Odds ratio against standard error in different genetic models).

T vs. C — Statistics with study removed

Study name	Point	Lower limit	Upper limit	Z-Value	p-Value
Yuliwulandari et al. 2016	1.292	1.108	1.507	3.269	0.001
Xiang et al. 2014	1.463	1.188	1.800	3.586	0.000
Singh et al. 2014	1.221	1.042	1.429	2.475	0.013
Santos et al. 2013	1.298	1.114	1.512	3.337	0.001
Gupta et al. 2013	1.200	1.025	1.406	2.262	0.024
Mishra et al. 2013	1.319	1.130	1.540	3.511	0.000
Ben Mahmoud et al. 2012	1.253	1.077	1.459	2.913	0.004
An et al. 2012	1.274	1.095	1.481	3.146	0.002
LV et al. 2012	1.273	1.094	1.483	3.113	0.002
Lee et al. 2010	1.276	1.097	1.484	3.162	0.002
Bozok et al. 2008	1.274	1.093	1.486	3.090	0.002
	1.278	1.109	1.484	3.210	0.001

Odds ratio (95% CI) with study removed

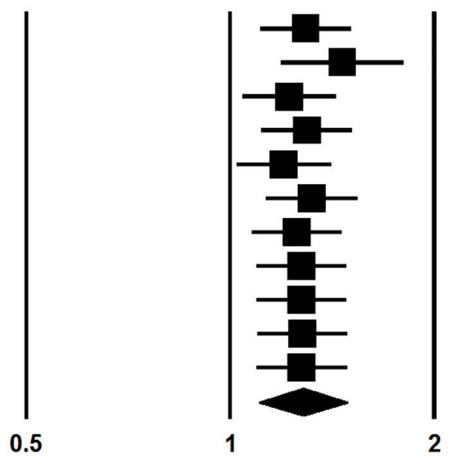

TT vs. CC — Statistics with study removed

Study name	Point	Lower limit	Upper limit	Z-Value	p-Value
Yuliwulandari et al. 2016	1.784	0.812	3.920	1.442	0.149
Xiang et al. 2014	2.327	1.198	4.521	2.494	0.013
Singh et al. 2014	1.489	0.686	3.233	1.007	0.314
Santos et al. 2013	1.840	0.795	4.258	1.425	0.154
Gupta et al. 2013	1.429	0.702	2.908	0.983	0.325
Mishra et al. 2013	1.985	0.967	4.075	1.869	0.062
Ben Mahmoud et al. 2012	1.585	0.711	3.532	1.127	0.260
Lee et al. 2010	1.688	0.782	3.643	1.333	0.183
Bozok et al. 2008	1.871	0.840	4.168	1.532	0.125
	1.751	0.844	3.631	1.506	0.132

Odds ratio (95% CI) with study removed

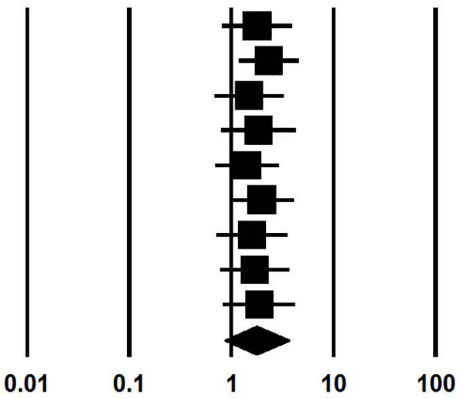

TC vs. CC — Statistics with study removed

Study name	Point	Lower limit	Upper limit	Z-Value	p-Value
Yuliwulandari et al. 2016	1.383	1.132	1.690	3.169	0.002
Xiang et al. 2014	1.402	1.049	1.874	2.283	0.022
Singh et al. 2014	1.325	1.084	1.619	2.742	0.006
Santos et al. 2013	1.401	1.151	1.706	3.360	0.001
Gupta et al. 2013	1.323	1.081	1.618	2.722	0.006
Mishra et al. 2013	1.365	1.117	1.668	3.038	0.002
Ben Mahmoud et al. 2012	1.355	1.114	1.647	3.044	0.002
An et al. 2012	1.349	1.109	1.641	2.990	0.003
LV et al. 2012	1.350	1.106	1.648	2.954	0.003
Lee et al. 2010	1.348	1.110	1.637	3.614	0.003
Bozok et al. 2008	1.324	1.086	1.615	2.772	0.006
	1.354	1.119	1.644	3.066	0.002

Odds ratio (95% CI) with study removed

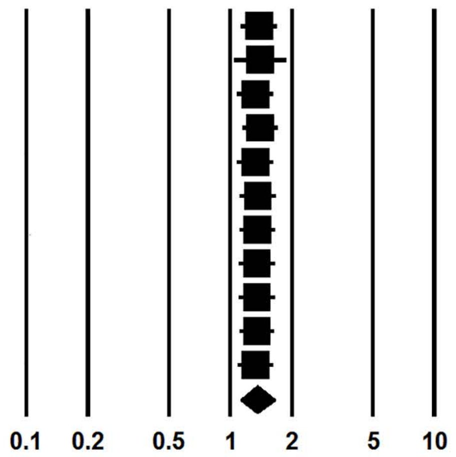

TT+TC vs. CC — Statistics with study removed

Study name	Point	Lower limit	Upper limit	Z-Value	p-Value
Yuliwulandari et al. 2016	1.370	1.129	1.661	3.192	0.001
Xiang et al. 2014	1.492	1.131	1.967	2.833	0.005
Singh et al. 2014	1.295	1.067	1.572	2.620	0.009
Santos et al. 2013	1.386	1.146	1.677	3.361	0.001
Gupta et al. 2013	1.283	1.057	1.559	2.515	0.012
Mishra et al. 2013	1.370	1.130	1.661	3.199	0.001
Ben Mahmoud et al. 2012	1.330	1.102	1.607	2.965	0.003
An et al. 2012	1.338	1.108	1.616	3.028	0.002
LV et al. 2012	1.340	1.106	1.622	2.992	0.003
Lee et al. 2010	1.339	1.110	1.614	3.058	0.002
Bozok et al. 2008	1.322	1.092	1.600	2.862	0.004
	1.344	1.115	1.620	3.103	0.002

Odds ratio (95% CI) with study removed

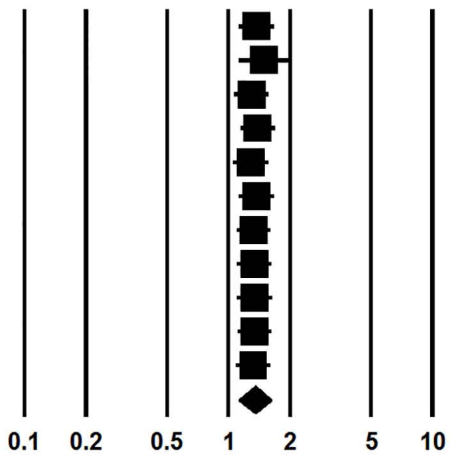

TT vs. TC + CC — Statistics with study removed

Study name	Point	Lower limit	Upper limit	Z-Value	p-Value
Yuliwulandari et al. 2016	1.482	0.774	2.838	1.186	0.236
Xiang et al. 2014	1.879	1.078	3.276	2.226	0.026
Singh et al. 2014	1.303	0.675	2.515	0.788	0.431
Santos et al. 2013	1.431	0.714	2.866	1.011	0.312
Gupta et al. 2013	1.255	0.689	2.285	0.742	0.458
Mishra et al. 2013	1.621	0.897	2.932	1.599	0.110
Ben Mahmoud et al. 2012	1.337	0.692	2.583	0.865	0.387
Lee et al. 2010	1.537	0.770	3.069	1.218	0.223
Bozok et al. 2008	1.594	0.833	3.048	1.409	0.159
	1.478	0.805	2.714	1.260	0.208

Odds ratio (95% CI) with study removed

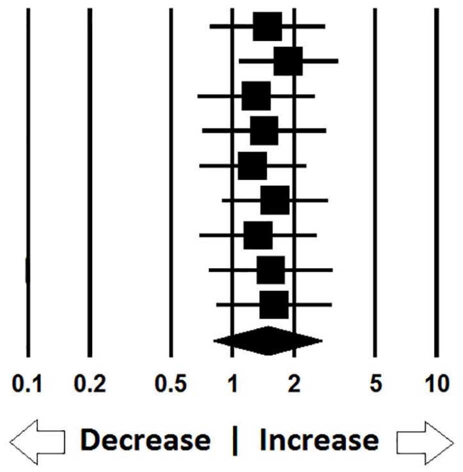

Figure SI4: Sensitivity analysis of NAT2 481C>T gene polymorphism with overall INH induced hepatotoxicity risk to evaluate the influence of each individual study on the pooled OR by deleting one single study each time for the overall analysis (for all the genetic models). Black square represents the value of OR and the size of the square indicates the inverse proportion relative to its variance. Horizontal line is the 95% CI of OR.

A vs. G — Statistics with study removed

Study name	Point	Lower limit	Upper limit	Z-Value	p-Value
Yuliwulandari et al. 2016	1.398	1.097	1.781	2.712	0.007
Xiang et al. 2014	1.469	1.128	1.913	2.851	0.004
Singh et al. 2014	1.496	1.193	1.876	3.492	0.001
Santos et al. 2013	1.430	1.138	1.797	3.074	0.002
Gupta et al. 2013	1.485	1.173	1.880	3.290	0.001
Mishra et al. 2013	1.362	1.087	1.706	2.686	0.007
Ben Mahmoud et al. 2012	1.430	1.131	1.807	2.990	0.003
An et al. 2012	1.378	1.090	1.742	2.677	0.007
LV et al. 2012	1.495	1.182	1.892	3.352	0.001
Lee et al. 2010	1.421	1.116	1.809	2.849	0.004
Kim et al. 2009	1.353	1.083	1.691	2.662	0.008
Bozok et al. 2008	1.350	1.089	1.675	2.731	0.006
	1.421	1.137	1.776	3.087	0.002

Odds ratio (95% CI) with study removed

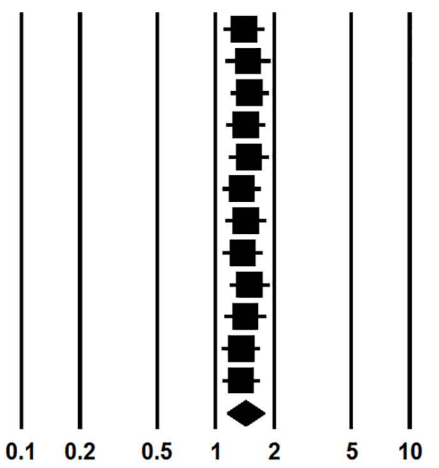

AA vs. GG — Statistics with study removed

Study name	Point	Lower limit	Upper limit	Z-Value	p-Value
Yuliwulandari et al. 2016	1.879	1.085	3.254	2.250	0.024
Xiang et al. 2014	2.182	1.208	3.942	2.585	0.010
Singh et al. 2014	2.251	1.365	3.710	3.181	0.001
Santos et al. 2013	2.023	1.191	3.435	2.609	0.009
Gupta et al. 2013	2.235	1.315	3.798	2.971	0.003
Mishra et al. 2013	1.887	1.101	3.236	2.309	0.021
Ben Mahmoud et al. 2012	1.941	1.141	3.300	2.447	0.014
An et al. 2012	1.825	1.084	3.071	2.265	0.024
LV et al. 2012	2.181	1.244	3.823	2.721	0.007
Lee et al. 2010	1.994	1.139	3.491	2.414	0.016
Kim et al. 2009	1.806	1.076	3.032	2.238	0.025
Bozok et al. 2008	1.781	1.084	1.926	2.277	0.023
	1.991	1.194	3.319	2.640	0.008

Odds ratio (95% CI) with study removed

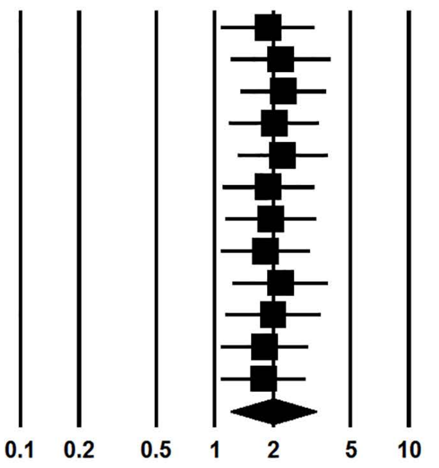

AG vs. GG — Statistics with study removed

Study name	Point	Lower limit	Upper limit	Z-Value	p-Value
Yuliwulandari et al. 2016	1.377	1.154	1.642	3.555	0.000
Xiang et al. 2014	1.428	1.141	1.787	3.113	0.002
Singh et al. 2014	1.355	1.137	1.614	3.398	0.001
Santos et al. 2013	1.359	1.144	1.613	3.502	0.000
Gupta et al. 2013	1.362	1.141	1.625	3.425	0.001
Mishra et al. 2013	1.305	1.097	1.554	3.002	0.003
Ben Mahmoud et al. 2012	1.378	1.159	1.637	3.638	0.000
An et al. 2012	1.355	1.134	1.620	3.336	0.001
LV et al. 2012	1.447	1.206	1.736	3.978	0.001
Lee et al. 2010	1.374	1.153	1.636	3.554	0.001
Kim et al. 2009	1.323	1.108	1.580	3.089	0.002
Bozok et al. 2008	1.334	1.122	1.568	3.254	0.001
	1.363	1.149	1.618	3.552	0.000

Odds ratio (95% CI) with study removed

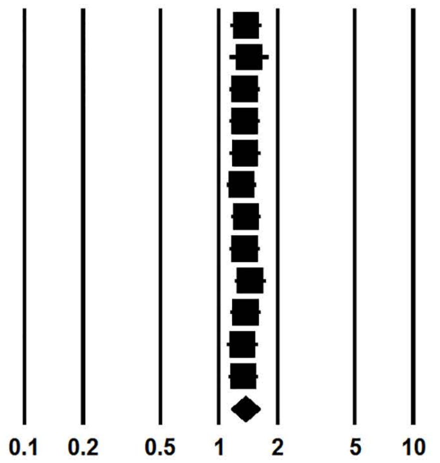

AA+AG vs. GG — Statistics with study removed

Study name	Point	Lower limit	Upper limit	Z-Value	p-Value
Yuliwulandari et al. 2016	1.394	1.181	1.647	3.919	0.001
Xiang et al. 2014	1.513	1.225	1.868	3.840	0.001
Singh et al. 2014	1.415	1.200	1.669	4.128	0.001
Santos et al. 2013	1.403	1.193	1.649	4.105	0.001
Gupta et al. 2013	1.420	1.202	1.677	4.128	0.001
Mishra et al. 2013	1.349	1.145	1.589	3.583	0.001
Ben Mahmoud et al. 2012	1.411	1.199	1.660	4.150	0.001
An et al. 2012	1.372	1.159	1.623	3.683	0.001
LV et al. 2012	1.489	1.255	1.768	4.552	0.001
Lee et al. 2010	1.404	1.190	1.656	4.019	0.001
Kim et al. 2009	1.347	1.139	1.592	3.488	0.001
Bozok et al. 2008	1.364	1.158	1.607	3.718	0.001
	1.402	1.193	1.647	4.111	0.001

Odds ratio (95% CI) with study removed

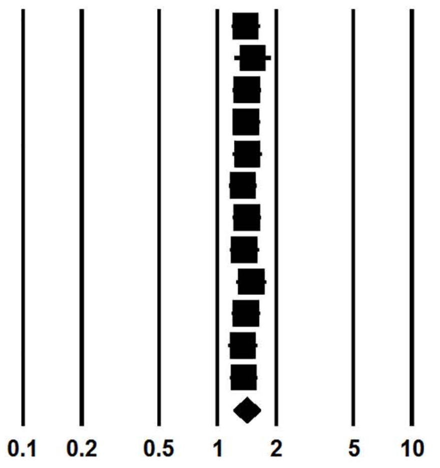

AA vs. AG+GG — Statistics with study removed

Study name	Point	Lower limit	Upper limit	Z-Value	p-Value
Yuliwulandari et al. 2016	1.513	0.915	2.501	1.614	0.107
Xiang et al. 2014	1.767	1.009	3.093	1.993	0.046
Singh et al. 2014	1.848	1.181	2.891	2.686	0.007
Santos et al. 2013	1.654	1.009	2.713	1.995	0.046
Gupta et al. 2013	1.830	1.123	2.984	2.424	0.015
Mishra et al. 2013	1.659	0.981	2.804	1.889	0.059
Ben Mahmoud et al. 2012	1.575	0.964	2.574	1.812	0.070
An et al. 2012	1.500	0.926	2.430	1.648	0.099
LV et al. 2012	1.740	1.023	2.961	2.042	0.041
Lee et al. 2010	1.614	0.959	2.717	1.802	0.071
Kim et al. 2009	1.502	0.922	2.446	1.636	0.102
Bozok et al. 2008	1.499	0.931	2.416	1.664	0.096
	1.637	1.015	2.641	2.022	0.043

Odds ratio (95% CI) with study removed

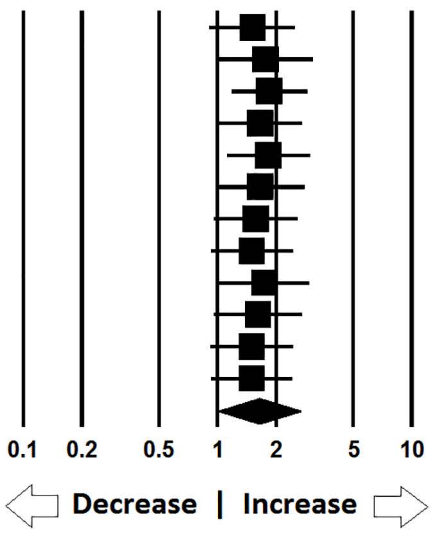

Figure SI5: Sensitivity analysis of NAT2 590G>A gene polymorphism with overall INH induced hepatotoxicity risk to evaluate the influence of each individual study on the pooled OR by deleting one single study each time for the overall analysis (for all the genetic models). Black square represents the value of OR and the size of the square indicates the inverse proportion relative to its variance. Horizontal line is the 95% CI of OR.

A vs. G

Study name	Point	Lower limit	Upper limit	Z-Value	p-Value
Yuliwulandari et al. 2016	1.426	1.023	1.986	2.096	0.036
Xiang et al. 2014	1.525	1.204	1.931	3.501	0.000
Santos et al. 2013	1.340	0.998	1.800	1.948	0.051
Mishra et al. 2013	1.378	1.011	1.878	2.026	0.043
Ben Mahmoud et al. 2012	1.426	1.054	1.931	2.299	0.021
An et al. 2012	1.396	1.003	1.944	1.979	0.048
LV et al. 2012	1.508	1.076	2.112	2.387	0.017
Lee et al. 2010	1.308	0.980	1.747	1.824	0.068
Kim et al. 2009	1.473	1.057	2.053	2.289	0.022
Bozok et al. 2008	1.350	1.008	1.809	2.011	0.044
	1.411	1.052	1.894	2.296	0.022

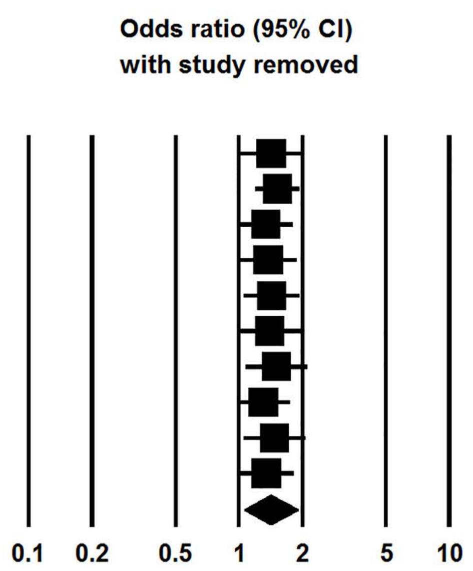

AA vs. GG

Study name	Point	Lower limit	Upper limit	Z-Value	p-Value
Yuliwulandari et al. 2016	1.192	0.964	1.474	1.625	0.104
Xiang et al. 2014	1.490	1.146	1.938	2.974	0.003
Santos et al. 2013	1.186	0.965	1.456	1.623	0.105
Mishra et al. 2013	1.166	0.948	1.434	1.456	0.145
Ben Mahmoud et al. 2012	1.213	0.991	1.486	1.872	0.061
An et al. 2012	1.119	0.903	1.387	1.025	0.305
LV et al. 2012	1.226	0.986	1.524	1.837	0.066
Lee et al. 2010	1.233	1.002	1.517	1.975	0.048
Kim et al. 2009	1.220	0.988	1.508	1.844	0.065
Bozok et al. 2008	1.184	0.965	1.452	1.622	0.105
	1.211	0.989	1.492	1.853	0.064

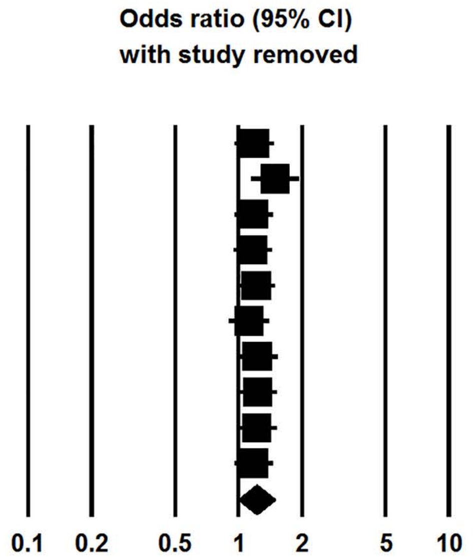

AG vs. GG

Study name	Point	Lower limit	Upper limit	Z-Value	p-Value
Yuliwulandari et al. 2016	1.192	0.964	1.474	1.625	0.104
Xiang et al. 2014	1.490	1.146	1.938	2.974	0.003
Santos et al. 2013	1.186	0.965	1.456	1.623	0.105
Mishra et al. 2013	1.166	0.948	1.434	1.456	0.145
Ben Mahmoud et al. 2012	1.213	0.991	1.486	1.872	0.061
An et al. 2012	1.119	0.903	1.387	1.025	0.305
LV et al. 2012	1.226	0.986	1.524	1.837	0.066
Lee et al. 2010	1.233	1.002	1.517	1.975	0.048
Kim et al. 2009	1.220	0.988	1.508	1.844	0.065
Bozok et al. 2008	1.184	0.965	1.452	1.622	0.105
	1.211	0.989	1.492	1.853	0.064

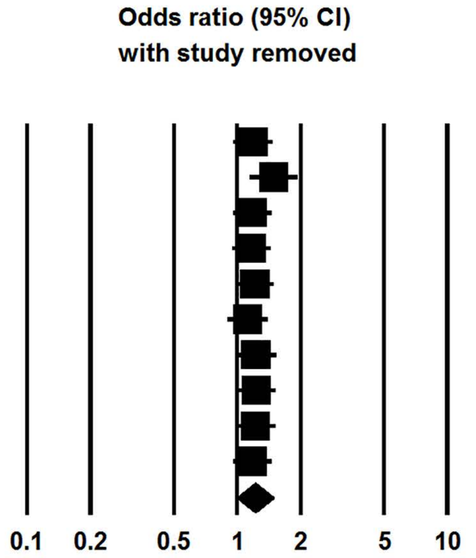

AA+AG vs. GG

Study name	Point	Lower limit	Upper limit	Z-Value	p-Value
Yuliwulandari et al. 2016	1.202	0.979	1.476	1.755	0.079
Xiang et al. 2014	1.548	1.203	1.990	3.403	0.001
Santos et al. 2013	1.191	0.975	1.454	1.716	0.086
Mishra et al. 2013	1.186	0.971	1.450	1.669	0.095
Ben Mahmoud et al. 2012	1.226	1.007	1.492	2.032	0.042
An et al. 2012	1.142	0.926	1.407	1.242	0.214
LV et al. 2012	1.251	1.013	1.544	2.079	0.038
Lee et al. 2010	1.200	0.980	1.470	1.761	0.078
Kim et al. 2009	1.233	1.005	1.514	2.006	0.045
Bozok et al. 2008	1.194	0.980	1.457	1.756	0.079
	1.223	1.005	1.498	2.014	0.044

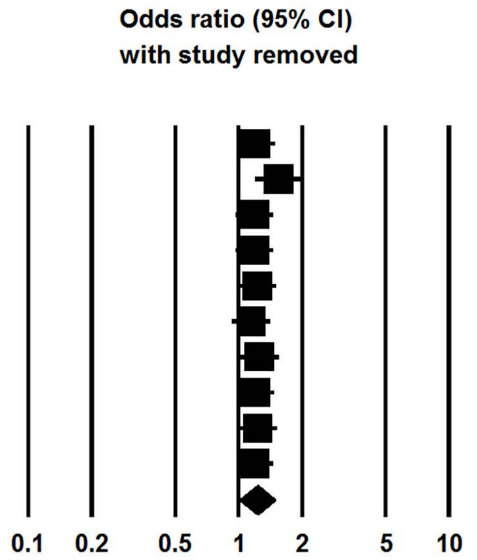

AA vs. AG+GG

Study name	Point	Lower limit	Upper limit	Z-Value	p-Value
Yuliwulandari et al. 2016	1.305	0.637	2.674	0.728	0.467
Xiang et al. 2014	1.793	0.855	3.760	1.547	0.122
Santos et al. 2013	1.208	0.585	2.492	0.511	0.610
Mishra et al. 2013	1.420	0.696	2.898	0.964	0.335
An et al. 2012	1.747	0.750	4.068	1.293	0.196
LV et al. 2012	1.588	0.760	3.317	1.231	0.218
Lee et al. 2010	1.011	0.484	2.109	0.029	0.977
Kim et al. 2009	1.421	0.688	2.934	0.949	0.343
Bozok et al. 2008	1.349	0.658	2.763	0.818	0.414
	1.396	0.697	2.797	0.042	0.346

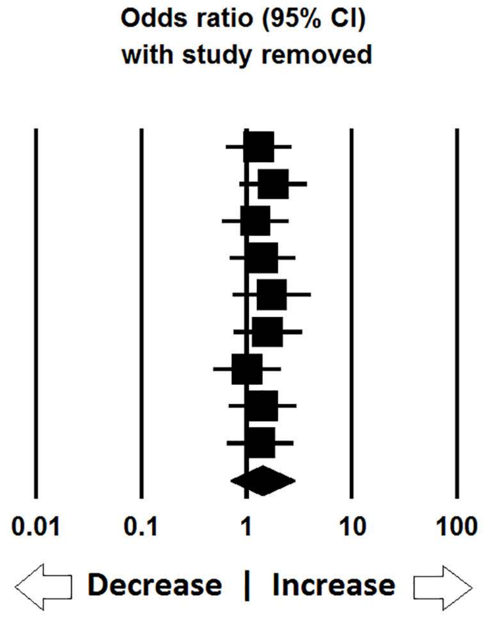

Figure SI6: Sensitivity analysis of NAT2 857G>A gene polymorphism with overall INH induced hepatotoxicity risk to evaluate the influence of each individual study on the pooled OR by deleting one single study each time for the overall analysis (for all the genetic models). Black square represents the value of OR and the size of the square indicates the inverse proportion relative to its variance. Horizontal line is the 95% CI of OR.
